# A systematic review of the relationship between muscle oxygen dynamics and energy rich phosphates. Can NIRS help?

**DOI:** 10.1186/s13102-024-00809-5

**Published:** 2024-01-20

**Authors:** Kevin Maliszewski, Andri Feldmann, Kevin K. McCully, Ross Julian

**Affiliations:** 1 Department of Neuromotor Behavior and Exercise, Institute of Sport and Exercise Sciences, University of Münster, Münster, 48149 Germany; 2 Institute of Sport Science, University of Bern, Bern, Switzerland; 3 Department of Kinesiology, University of Georgia, Athens, USA; 4 School of Sport and Exercise, University of Gloucestershire, Cheltenham, England

**Keywords:** Near-infrared spectroscopy, NIRS, Energy rich phosphates, PCr, Muscle oxygen dynamics, Oxidative capacity, Arterial occlusion, Exercise, Muscle oxygenation

## Abstract

**Background:**

Phosphocreatine dynamics provide the gold standard evaluation of in-vivo mitochondrial function and is tightly coupled with oxygen availability. Low mitochondrial oxidative capacity has been associated with health issues and low exercise performance.

**Methods:**

To evaluate the relationship between near-infrared spectroscopy-based muscle oxygen dynamics and magnetic resonance spectroscopy-based energy-rich phosphates, a systematic review of the literature related to muscle oxygen dynamics and energy-rich phosphates was conducted. PRISMA guidelines were followed to perform a comprehensive and systematic search of four databases on 02-11-2021 (PubMed, MEDLINE, Scopus and Web of Science). Beforehand pre-registration with the Open Science Framework was performed. Studies had to include healthy humans aged 18–55, measures related to NIRS-based muscle oxygen measures in combination with energy-rich phosphates. Exclusion criteria were clinical populations, laboratory animals, acutely injured subjects, data that only assessed oxygen dynamics or energy-rich phosphates, or grey literature. The Effective Public Health Practice Project Quality Assessment Tool was used to assess methodological quality, and data extraction was presented in a table.

**Results:**

Out of 1483 records, 28 were eligible. All included studies were rated moderate. The studies suggest muscle oxygen dynamics could indicate energy-rich phosphates under appropriate protocol settings.

**Conclusion:**

Arterial occlusion and exercise intensity might be important factors to control if NIRS application should be used to examine energetics. However, more research needs to be conducted without arterial occlusion and with high-intensity exercises to support the applicability of NIRS and provide an agreement level in the concurrent course of muscle oxygen kinetics and muscle energetics.

**Trial registration:**

https://osf.io/py32n/.

**Key points:**

1. NIRS derived measures of muscle oxygenation agree with gold-standard measures of high energy phosphates when assessed in an appropriate protocol setting.

2. At rest when applying the AO protocol, in the absence of muscle activity, an initial disjunction between the NIRS signal and high energy phosphates can been seen, suggesting a cascading relationship.

3. During exercise and recovery a disruption of oxygen delivery is required to provide the appropriate setting for evaluation through either an AO protocol or high intensity contractions.

## Introduction

Mitochondria are complex filamentous organelles that are the primary structures responsible for cellular energetics [1, 2]. Mitochondria use electrons from substrate oxidation to shift protons within the electron transport system, causing a chemiosmotic gradient and driving ATP production as an energy currency. The last electron acceptor is oxygen, concluding that there is no substrate oxidation and no energy flux during energy demands without oxygen [3, 4]. Near-infrared spectroscopy (NIRS) can be used in vivo as a non-invasive and simple assessment to measure the oxygen consumption rate in skeletal muscle [5–7].

As shown, after muscle activity or ischemic states, phosphocreatine (PCr) recovery depends on oxidative ATP production by mitochondria [8–10]. Consequently, the dependence of PCr recovery on oxidative ATP production and overall oxygen availability may indicate a close correlation between oxygen availability and energy-rich phosphates. During activity, ATP levels in the muscle are buffered through PCr, dynamically depleted and synthesized through creatine kinase. Under high ATP demands, mitochondrial respiration is stimulated by rising ADP concentrations, reflected by PCr concentrations in the muscle [11, 12]. Assuming creatine kinase equilibrium, the recovery rate of PCr after exercise represents mitochondrial ATP production [13, 14]. These changes can be measured via phosphorus nuclear magnetic resonance spectroscopy (*P*-MRS) and provide quantifiable information about phosphocreatine, ATP, and inorganic phosphate (Pi). Overall, mitochondrial function [15, 16] and a higher oxidative capacity [17–20] are associated with enhanced performance, from patients with chronic illness to endurance athletes. Since magnetic resonance spectroscopy (MRS) is expensive, limited in availability, and complex movements of healthy individuals are not feasible, NIRS has garnered more interest in assessing oxidative capacity, comparing muscle oxygen kinetics and changes in energy-rich phosphates [6, 7]. Moreover, muscle oxygen kinetics are increasingly measured in both laboratory and applied sports settings to assess muscle performance and training status [21]. However, studies regarding oxygen dynamics differ in their approaches and show contradictory results that do not consistently demonstrate the connection between muscle oxygen dynamics and energy-rich phosphates [6, 7, 21, 22].

Perrey & Ferrari [21] previously conducted a systematic review on muscle oximetry in sports science, presenting the development of the application of oximetry in sports during the past 35 years. The authors highlighted the need for a more in-depth comparison of physiological parameters to show the advantages of the routine use of muscle oximetry [21]. Nonetheless, Campbell and Marcinek [23] discussed nuclear MRS and optical measurements for in vivo evaluations of mitochondrial biogenetics in their narrative review and provided an overview of some possible approaches. The authors submitted that NIRS could represent an alternative to *P*-MRS for measuring mitochondrial capacity in skeletal muscle and emphasize this novel approach’s development, validation, and application.

A better and more comprehensive understanding of the relationship between corresponding muscle oxygen values measured by NIRS and energy-rich phosphates during rest, activity, or ischemic assessments will be helpful in the development of non-invasive continuous examinations of integrated energetics in athletes.

A preliminary literature search showed that there is currently no systematic summary regarding the relationship between muscle oxygenation measured by NIRS and phosphate synthesis and resynthesis. This review will favor a practical perspective on an integrated and dynamic evaluation of energetics and analyses of oxygenation data solely from muscle oxygen kinetics and phosphate-related measurement methods. Therefore, this review examines how muscle oxygenation data measured by NIRS represents energy-rich phosphates according to published research to address whether NIRS can be useful in determining energy-rich phosphates. As will become evident, apart from a systematic literature review the results are narrative in nature, as large methodical and technical variation makes any systemic analysis or modeling difficult.

## Methods

The study was conducted according to the Preferred Reporting Items for Systematic Reviews and Meta-Analysis (PRISMA) statement [24]. The protocol was pre-registered on the Open Science Framework before full searches and analysis (https://osf.io/py32n). Since the current study reviewed published studies, ethical approval or patient consent was unnecessary.

### Search strategy and study identification

Literature was searched in PubMed/MEDLINE, Scopus, and Web of Science. All searches were conducted on February 11, 2021. The Boolean method was used with the operators AND/OR/NOT to limit the results to relevant studies containing the following search terms: “near-infrared spectroscopy”, “NIRS”, “SmO2”, “HHb”, “O2Hb”, “TSI”, “mVO2” or “tHb” in combination with “phosphate”, “phosphorus”, “creatine phosphate”, “phosphocreatine”, “adenosinetriphosphate”, “adenosine triphosphate”, and “phosph*”. Reference lists from the selected studies were screened for other relevant studies; duplicates and studies known to the authors were also included. Two authors (KM and AF) have independently screened the titles and abstracts for relevance. The two mentioned authors examined the remaining full texts based on the inclusion and exclusion criteria. In the event of discrepancies arising, a third author (RJ) provided an examination of the relevant articles to reach a consensus decision. The detailed search strategies for each database are available in a supplementary file (Supplemental material, https://osf.io/7u4ym/?view_only=1048d187f51c4cdb970bb6dc6632738d).

### Selection criteria

#### Inclusion

Studies eligible for the present review article had to [1] be published in the English language, [2] have abstracts available for screening, [3] include relevant data concerning the relationship of oxygenation data and energy-rich phosphates, [4] include human subjects at age 18–55 years. There was no limitation to publication date since the first NIRS studies were published in 1977 and contributed to the understanding of the development of NIRS measurements [25].

#### Exclusion

Studies were excluded if one of the following criteria were met: (1) acute injured and other clinical population, (2) laboratory animals, (3) in vitro or in situ experiments (muscle biopsies, high-resolution respirometry, measuring enzymatic activity), (4) data only assessing NIRS, (5) dietary intervention, (6) non-research letters and editorials, case studies, case series, grey literature - such as theses and dissertations -, abstracts or congress communications were excluded, as well as epidemiological, commentaries, literature, narrative, and systematic reviews.

### Data extraction and quality assessment

One author (KM) extracted the relevant data from each included study using predesigned table forms on Microsoft Excel. A second author (AF) confirmed the results. Discrepancies between authors were resolved by the third author (RJ). For examining the methodological quality of all included studies, the Effective Public Health Practice Project Quality Assessment Tool (EPHPP) (Armijo-Olivo et al., 2012) was used and performed independently by two reviewers (KM & AF). The EPHPP assess the analytical cross-sectional and intervention studies, which will include the comparisons of the measurement tools. All questions had to be rated as strong (3 points), moderate (2 points), or weak (1 point), and domain scores were averaged to provide the total score. The maximum total score per study was 3.00. Based on their total score, studies were assigned a quality rating of weak (1.00–1.50), moderate (1.51–2.50) or strong (2.51–3.00).

## Results

### Study selection

**Fig. 1:**
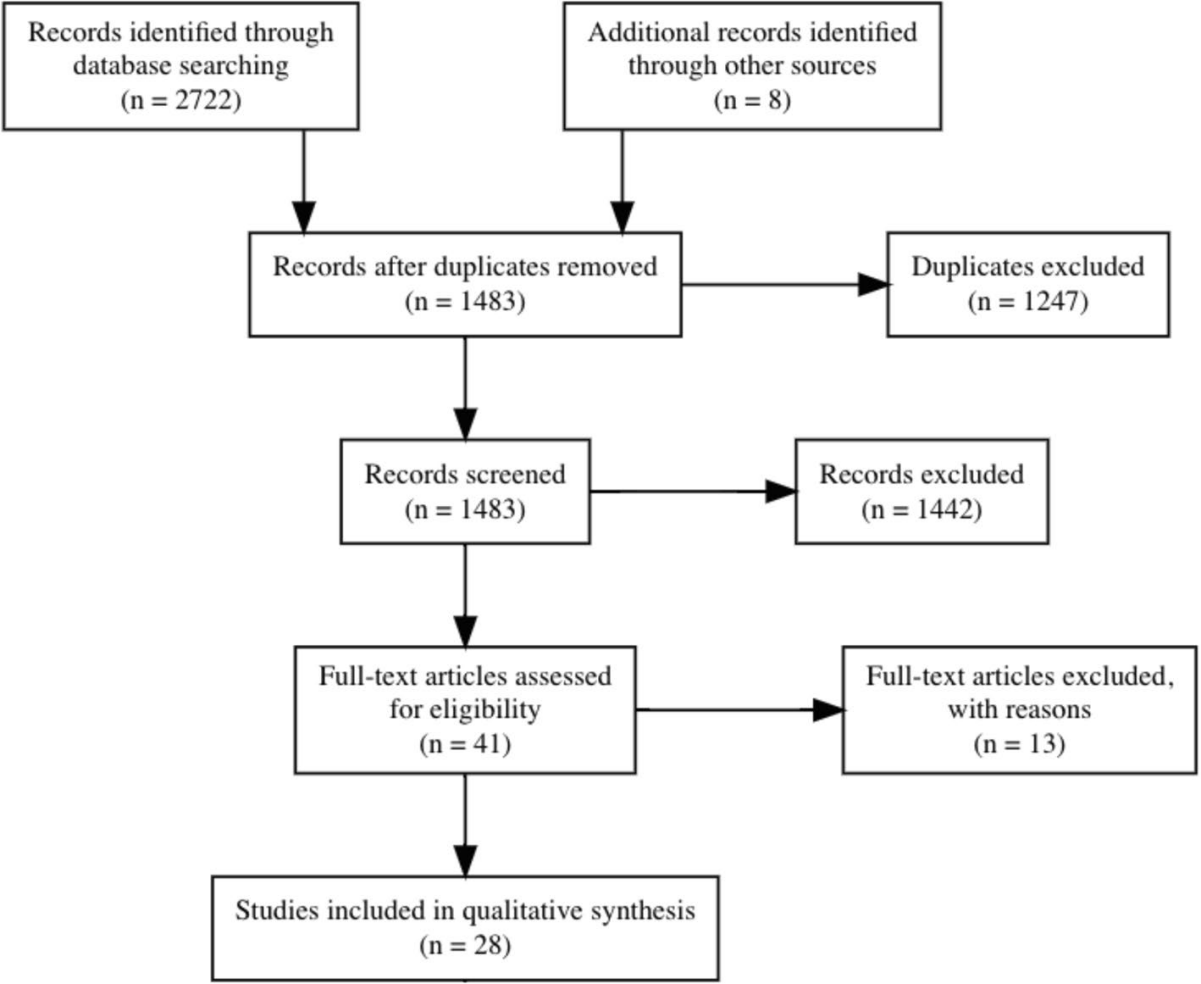
PRISMA flow diagram of the selection process of the journal articles included in the systematic review

As shown in Fig. 1, 2722 records were identified in the four electronic databases (MEDLINE/Pubmed, Scopus, and Web of Science). One thousand two hundred forty-seven duplicates were removed, and 1483 remained for screening. Forty-one studies were considered relevant after assessing the title and abstract inspection. Full-text screening showed 28 articles included in the systematic review (Fig. 1).

### Study characteristics

The main characteristics of included studies are presented in Table 1. Studies were published between 1994 and 2020. Exercise intensity in the studies varied from resting to maximal intensity. Muscles of the lower and upper extremities and lumbar were included. Moreover, arterial occlusion (AO) was applied in 15 studies [6, 26, 28–30, 32, 34–36, 38, 45, 49, 50, 52]. Two studies included data on ischemia in the absence of exercise [28, 29]. All other studies involve training and/or post-exercise recovery. The study populations were heterogeneous, ranging from sedentary to highly active participants. The sample size ranged from four to 50 participants.

**Table 1:** Summary of findings from studies investigating muscle oxygen saturation and energy rich phosphates

Authors & Year	Subjects, Age, and Sex	Exercise protocol	pH Value	Duration & Intensity	Muscle	Devices & Variables	Results
McCully et al.,1994 [22]	5, 36 ± 13, male	Two submaximal and two rapid (maximal) plantar flexion exercises were performed	Submaximal bouts: > 7.0, Maximal bouts: < 6.8	Two 5 min bouts and two 1 min bouts. First two bouts were performed with PCr depletion to 60% of resting values, then two bouts were performed till PCr values reached 5–20% of resting values.	Gastrocnemius and soleus muscle	Measured with a homebuilt spectrometer system (PCr; Pi); Runman (HbO_2_)	Rate of recovery was slower for PCr than for HbO_2_ in maximal exercise. The maximal PCrTc value (68.3 ± 10.5 s) was significantly higher than the submaximal PCrTc values (36 ± 6.5 s) and both the submaximal and maximal HbTc values (27.6 ± 6 and 29.4 ± 5.5 s).
Hamaoka et al., 1996 [26]	5,25–31, male	1) AO for the measurement of resting metabolic rates; 2) measurement of oxidative metabolism during isotonic hand-grip contractions; 3) AO was used at the end of exercise to distinguish between O_2_ supply and O_2_ demand	–	10, 20, 30, and 40% MVC. 5 min each intensity, 1 contraction every 4 s.	Flexor digitorum superficialis	Otsuka Electronics (PCr, Pi, ADP); HEO-100, OMRON Inc. (VO_2mus_)	Averaged VO_2mus_ and ADP values during muscle contractions were correlated (*r2* = .98, *p* > .01), VO_2mus_ and PCr were correlated (r2 = .99, *p* > .01).
Yoshida et al., 1996 [27]	6, 19 ± .4, male	Knee flexion exercises with active and passive recovery. Exercise test consisted of ramp test to obtain the relative work rate, submaximal repeated exercise for passive recovery and submaximal interval exercise for active recovery.	Low- and high-pH groups (no values).	Exercise intensity was selected as 60% of maximal exercise intensity attained in the ramp exercise test. 1) Ramp test: 1 min of resting measurement, exercise intensity was increased by 0.41 W every 15 s until volitional exhaustion. 2) Passive recovery test consisted of 2 min of exercise and 2 min passive recovery, 2 min exercise, followed by 5 min recovery. 3) Active recovery test consisted of 2 min unloaded warm-up, 2 min of exercise, followed by 2 min of unloaded exercise. Procedure was repeated, followed by a resting recovery period of 5 min.	Biceps femoris muscle	EX90, JEOL, Japan (PCr); Hamamatsu, NIR-4 s (Hb-Mb: Total hemoglobin/myoglobin)	PCr time constant during active recovery appeared a little slower than during passive recovery (30.2 and 32.1 vs. 25.3 and 25.9). Hb-Mb returned to baseline only during passive recovery (no values).
Binzoni et al., 1997 [28]	8, −, male	Three bouts of 5 min ischemia and 5 min reperfusion in a supine position.	The pH value decreased (.02 pH).	10 min each cycle. Full ischemia without movement was conducted.		SMIS, Guilford, UK (PCr); NIRO 500 Hamamatsu Photonics, Japan (Hb, HbO_2_, Hb_tot_)	No changes in PCr and ATP concentration were found. Hb, HbO_2_ and Hb_tot_ changed during ischemic preconditioning.
Binzoni et al., 1998 [29]	50, 29.7 ± 4–37 ± 12, 20 females, 30 males	Group 1: 5 min measurement of resting baseline while the subject was seating; Group 2: 5 min of resting baseline, following a sequence of three cycles, each consisting of 5-min ischemia followed by 5-min reperfusion. Subject was lying supine; Group 3: Same protocol as for Group 2 with Na-NMRS. In addition, control NA-NMRS measurements were carried out in separate sessions before the intervention protocol.	Increase (0.02 ± .004) observed at the end of the exercise protocol and the baseline values.	Group 1: 5 min after an initial 5-min readjustment period; Group 2: 5 min resting, three cycles of 5-min ischemia and 5-min reperfusion; Group 3: As group 2. Additionally, Na-NMR spectra were acquired 10 min after the lying position was performed.	Gastrocnemius muscle	SMIS, Guilford, UK (PCr); NIRO 500 Hamamatsu Photonics, Japan (Hb, HbO_2_, Hb_tot_)	No concentration changes in PCr occur over time when contraction is not present. A constant de-crease in ∆HbO_2_ is accompanied by an increase in ∆Hb. Hb_tot_ varies among subjects.
Boushel et al., 1998 [30]	7, 25–39, male	Rhythmic handgrip was performed at a cadence of 1 Hz.	The pH value ranged from 7.38 to 6.8 at 15% MVC to 6.38 at 30% MVC.	Experiment was conducted at 15 and 30% MVC. Exercise duration was 2 min. 5 s before the end of exercise, the occlusion cuff was inflated and maintained for 10 min. After releasing cuff pressure 20 min of recovery followed and the protocol was repeated at 30% MVC.	Forearm flexor muscle	Vivospec spectrometer, Otsuka Electronics and Magnex Scientific, Abingdon, UK (PCr); INVOS 3100, Somanetic Corporation, Troy, MI and NIRO 500, Hamamatsu, Japan (NIRS-O_2_)	NIRS-O_2_-derived rate of metabolism in forearm flexor muscles highly correlated with muscle metabolic rate evaluated by MRS-derived indices of ATP turnover. MRS-derived and NIRS-derived indices of muscle metabolism showed an approximate twofold increase at 15% MVC compared to 30% MVC.
Kutsuzawa et al., 2001 [31]	9, 29 ± 5, 1 female, 8 male,	Repetitive hand gripping of a lever at the rate of 20 grips per minute.	The pH value dropped from 7.05 ± .08 at rest to 6.85 ± .16 at the completion of the exercise phase	7% of the maximum grip strength of the non-dominant arm. 3 min exercise.	Forearm flexor muscle	BEM 250/80 and Otsuka Electronics Co., Osaka, Japan (PCr); OM-100A, Shimadzu Co. Kyoto, Japan (deoxy-Hb, oxy-Hb)	At rest: PCr/(PCr + Pi) .855 ± .025; at exercise end: PCr/(PCr + Pi) .582 ± .104; Deoxy-Hb increased in a monoexponential fashion and reached a plateau, oxy-Hb was a direct reflection of this behavior.
Sako et al., 2001 [32]	12, 25 ± 5, male	Two bouts rhythmic handgrip exercise were performed to measure independently *P*-MRS and NIRS. One session was conducted to measure resting metabolic rate.	The pH value was 7.02 ± .01 at rest and decreased to 6.7 ± .19 at the end of exercise. At 30 s after exercise pH dropped to 6.61 ± .3.	Experiment was conducted at 12% (*n* = 3),18% (*n* = 3), or 24% (*n* = 6) of MVC. Resting metabolic rate was measured during 15 min of arterial occlusion. Before each bout of exercise, subjects underwent arterial occlusion for 1 min followed by 2 min of resting period. Each exercise was conducted for 3 min	Forearm flexor muscles	Otsuka Electronic Co. Ltd., Osaka (PCr); OM-100A, Shimadzu (Oxyhemoglobin)	Muscle oxidative metabolic rate at 30 s postexercise was evaluated from the ratio of the rate of oxyhemoglobin decline during occlusion and the rate at rest. PCr resynthesis rate and NIRS-related muscle oxidative metabolic rate at 30 s after exercise correlated significantly (r = .965, *p* < .001).
Hamaoka et al., 2003 [33]	5, 24–36, male	Subjects performed submaximal grip exercise.	The pH value was not measured.	5 sec isometrics and 5 sec relaxation. After 1 min of resting arterial occlusion, 2 min of recovery followed, and thereafter exercise was performed for 3 min at 50% MVC.	Flexor digitorum superficialis muscle	Otsuka Electronics Inc. (PCr); HEO-200, OMRON Inc. Japan (Oxygenated, deoxygenated, and total hemoglobin)	Without AO similar kinetics between rate of deoxygenation and PCr concentration at the onset of exercise were observed. Correlation between the rate of deoxygenation and PCr concentration at the onset of exercise (*r* = .96, *p* < .05) were found.
Kime et al., 2003 [34]	7, 29 ± 3, male	Subjects performed a maximal isometric handgrip exercise for 10s and the arm was completely relaxed immediately after exercise.	Minimum intracellular pH 6.85.	Exercise duration was at 10 sec. Intensity was maximal. Reoxy-rate measurement was extended to 60s.		Otsuka Electronic Co. Ltd., Osaka (PCr); OM-100A, Shimadzu (Oxy-Hb)	Reoxy-rate and PCr time constant showed significant positive correlation (*r2* = .939, *p* < .001).
Kime et al., 2003 [35]	7, 28 ± 3, male	Static hand grip exercise; initial 4 seconds was used for calculating reoxygenation rate	Minimum intracellular pH 6.84 ± .04.	10 seconds at 30, 60, and 90% MVC.		Otsuka Electronics (PCr); OM100A, Shimadzu, Kyoto, Japan (Oxy-Hb)	Reoxy-rate was correlated to the PCr time constant (*r* = 0.825, *p* < .05 at 90% MVC exercise). No significant results at 30 and 60% MVC exercises were found.
Nagasawa et al., 2003 [36]	8, 24–35, male	Two bouts of dynamic handgrip exercise with the same procedure to measure VO_2mus_ recovery after exercise with NIRS combined with arterial occlusion and to measure in a second session *P*-MRS in a magnet.	Minimum pH value during recovery in all subjects was above 6.95.	Duration was dependent of incremental test length. Intensity was increased by 5% of MVC every 2 min, starting from 10% MVC up to 20% MVC.	Flexor digitor-um superfi-cialis.	BEM 250/80; Otsuka Electronics Co., Osaka Japan (PCr); HEO200, Omron Inc., Japan (VO_2mus_)	VO_2mus_ was based on decline rate of oxygenated hemoglobin. Time constant for the VO_2mus_ in recovery periods and the Tc for PCr recovery were 33.1 ± 9 and 35 ± 8.5 s. The Tc for muscle oxygen consumption recovery correlated significantly with the Tc for PCr recovery (*r* = .92, *p* < .01).
Forbes et al., 2006 [37]	7, 24 ± 4, male	Rest measurement, single-leg plantar flexion exercise, and recovery in control (PETCO_2_ 33 mmHg) and hyperventilation (PETCO_2_ conditions.	pH mean drop of .07	20 min rest, 6 min exercise, and 10 min recovery. Exercise was conducted at 75% of pH threshold.	Lateral gastrocnemius	IMRIS, Winnipeg, MB, Canada and Surrey Medical Imaging Systems, Guilford, UK (PCr); Wright Instruments, Enfield, Middlesex, UK (HHb, HbO_2_, Hb_tot_))	Hyperventilation-induced hypocapnia, compared to control condition, was associated with 1) a slower time course and greater breakdown in PCr during the transition to moderate-intensity exercise and 2) a similar time course of adaptation of HHb but greater increase in HHb signal during the exercise transition.
Kimura et al., 2006 [38]	7, 24–30, male	The measurements of 1) resting metabolic rate using the arterial occlusion method, 2) exercise under intramuscular condition, and 3) exercise under anaerobic condition were performed. An isometric wrist flexion was performed at a constant load.	No differences in pH at each %MVC between condition 2) and 3).	1) After 2 min of rest, forearm arterial blood flow was arrested by a pneumatic cuff. AO was maintained for 12 min at rest. 2) Under intramuscular condition, exercise was performed just after the beginning of the arterial occlusion. 3) For anaerobic exercise, 6 min of resting arterial occlusion was carried out before starting with the exercise protocol. Exercise was performed with 1 min for 30 and 50% MVC, and until exhaustion for 70% MVC.	Flexor digitorum superficialis	Otsuka Electronic Co. Ltd., Osaka (PCr); HEO-200, Omron Co. Ltd., Kyoto (HbO_2_)	At 30% MVC, PCr breakdown of 2) was significant lower (*p* < .05) compared to 3). Oxidative metabolism using intramuscular O_2_ above 50% MVC did not contribute to the decrease in PCr breakdown. The availability of O_2_ at higher intensities was not enhanced in spite of the significantly increased ADP and adequate stored intramuscular O_2_.
Jones et al., 2008 [39]	6, 28 ± 6, male	Single-legged knee-extension exercise in a prone position	Shift of pH in first exercise bout: 7.05 ± .01 to 6.89 ± .12. It recovered to 6.97 ± .06. Shift of pH in second bout to 6.84 ± .22. The pH value was significant higher in the third minute of the second bout compared to the first bout.	Two 6 min bouts of exercise with 6 min of resting recovery in between. 80% of the peak work rate.	Quadriceps	Intera, Philips, Eindhoven, The Netherlands (PCr); NIRO 300, Hamamatsu Photonics KK, Japan (O_2_Hb, HHb, and Hb_tot_)	O_2_Hb and Hb_tot_ were significantly higher in the baseline period prior to the second compared with the first exercise bout. Priming exercise did not alter tau for PCr, indicating that muscle oxygen availability was not limiting the initial rate of PCr hydrolysis within the rest-to-exercise transient. PCr dynamics were not different between bouts (51 ± 8 s vs. 52 ± 17 s) but over the entire response were faster in the second bout (mean response time 72 ± 16 s vs. 57 ± 8 s), as a consequence of a greater decrease in PCr in the beginning phase and a reduction in the magnitude of the ‘slow component’ in PCr beyond 3 min of exercise (10 ± 6% vs. 5 ± 3%; *p* > .05).
Forbes et al., 2008 [40]	7, 24 ± 4, male	Three Single-leg plantar flexion exercise was performed. Dynamic contractions were performed at the rate of .5 Hz.	–	6 min of resting measurements, followed by two 6 min bouts of heavy-intensity exercise separated by either 3-,6-, or 15-min resting recovery. The order of the three experimental trials was randomized. Additionally, each condition was performed three times to improve the confidence of the parameter estimations for PCr kinetics. Work-rate was set between pH threshold and the maximum work-rate achieved in a previous ramp test.	Gastrocnemius	IMRIS, Winnipeg, MB, Canada and Surrey Medical Imaging Systems, Guilford, UK (PCr); Wright Instruments, Enfield, Middlesex, UK (HHb, Hb_tot_)	Hb_tot_ was elevated after 3 min and the HHb onset kinetics were slowed in the bout after 3 min recovery. PCr slow component was similarly reduced in all subsequent bouts. O_2_ delivery at exercise onset is not directly related to the time course of adaptation of PCr breakdown at the onset of subsequent bouts of heavy-intensity exercise.
Zange et al., 2008 [41]	20, 21 ± 3, male	3 min of plantar flexion isometric contraction at 40% of MVC, CON performed under an AO, 3 min of isometric contraction at 40% MVC superimposed by a 20 Hz VIB of the pedal, and VIB under AO.	At the end of isometric contraction, pH decreased by .09. Only under AO vibration caused a decrease in pH of .16.	1 min rest, 3 min exercise, 10 min recovery at rest, 3 min exercise, and further 10 min recovery at rest. Per examination only one period of VIB and one period of AO was performed. Intensity was set at 40% MVC.	Gastrocnemius	Bruker-Biospec 47/40, Bruker-Medical, Ettlingen, Germany (PCr, ATP); Oxymon, Artinis Medical Systems, Andelst, The Netherlands (HbO_2_)	The average level of ATP did not change during exercise and recovery. After 3 min of exercise muscles had consumed in average 27% of their initial PCr. Under AO, contraction with VIB caused a larger decrease in PCr of 35%. Under AO, contraction plus VIB caused a significantly decrease in PCr compared to contraction without VIB. Natural perfusion did not change the decrease in PCr.
Layec et al., 2009 [42]	15, 32 ± 6, 1 female, 14 males	Two-leg knee-extension exercise	The pH value was 7.02–7.08 at the beginning of the exercise sessions and 6.84–6.9 at end of exercise.	Two 6 min bouts of exercise with 6 min of resting recovery in between. Exercises were performed at 35% MVC. If slow phase of oxygen uptake kinetics did not occur, intensity was increased.	Vastus lateralis	Siemens Vision Plus system, Siemens AG, Erlangen, Germany (PCr, ATP); NIRO-300, Hamamatsu Photonics, Hamamatsu City, Japan (HHb)	A reduced deoxygenation time delay and an increased deoxygenation amplitude coupled to an increased oxidative ATP cost in the early part of exercise was observed. ATP production from PCr breakdown and glycolysis were reduced. The priming exercise increased the PCr Tc within the fundamental phase of the response but did not alter the subsequent PCr slow component.
Willcocks et al., 2010 [43]	11, 24 ± 4, 5 female, 6 males	Two constant work rate exercise tests.	There was no significant change in pH in men (.06 ± .07, *p* = .11) or women (.03 ± .04, *p* = .18)	Two minutes of resting data collection and seven minutes of work. Work was performed at an intensity corresponding to 20% of the difference between the workload at the intracellular P/PCr threshold and the maximal workload.	Rectus femoris	Intera, Philips, The Netherlands (PCr); NIRO-300, Hamamatsu Photonics KK (HHb)	6 adults had an upward HHb, 4 maintained a steady-state plateau and 1 displayed a falling HHb whereas PCr decreased from rest to end-exercise by 56 and 44% in men and women, respectively.
Layec et al., 2012 [44]	13, 30 ± 6, male	Two-leg knee-extension exercise. After 2 min resting period, a series of ‘step’ exercise tests to the work rate which induces slow phase of oxygen uptake kinetics.	Delta pH was 0.45 in sedentary and − 0.69 in trained population	One 6 min bout at 35% MVC.	Vastus lateralis	Siemens Vision Plus system; Siemens AG, Erlangen, Germany (PCr); NIRO-300; Hamamatsu Photonics, Hamamatsu, Japan (HbO_2_, HHb, Hb_tot_)	HbO_2_ increased progressively after 120 s. As a result of increased O_2_ availability, the PCr slow component can be minimized in athletes.
Layec et al., 2013 [45]	9, 23, male	Constant-load submaximal plantar flexion at 40% of MVC either under conditions of free flow or reactive hyperemia, induced by a period of brief ischemia during the last minute of exercise.	No significant change in pH Value	After 2 min of rest, subjects exercised for 5 min at 40% of MVC followed by 5 min of recovery.	Calf muscle	Tim-Trio, Siemens Medical Solutions (PCr); Oxiplex TS, ISS (HHb, TOI)	A release of brief circulatory occlusion at the offset of plantar flexion exercise 1) led to a substantially increased convective O_2_ delivery and capillary blood flow in muscle, 2) was associated with a faster tissue reoxygenation, and 3) increased muscle PCr synthesis and estimated peak mitochondrial respiration rates.
Ryan et al., 2013 [6]	16, 23 ± 3, 6 female, 10 males	A plantar flexion exercise was conducted, following 10–18 brief (5–10s) AO.	Minimum ph value during resting was 7.05 ± .03 and at end of exercise at 6.98 ± .06.	10 sec of plantar flexion was performed. Intensity was set with pneumatic resistance (psi) which allowed for a minimum of two contractions. Between bouts 5–7 min recovery period was added.	Gastrocnemius	GE Healthare, Waukesha, WI (PCr); Oxymon MKIII, Artinis Medical Systems (mVO_2_)	Recovery kinetics of mVO_2_ were not statistically different from and correlated well with recovery kinetics of PCr after short-duration exercise. Rate of recovery of mV measured by NIRS correlated well with the rate of recovery of PCR (Pearson’s *r* = .88, *p* < .001 for Channel 1 and Pearson’s *r* = .95, *p* < .001 for Channel 2).
Fulford et al., 2014 [46]	10, 19–25, 5 female, 5 male	Participants were asked to maintain their back extended in a prone position until the upper body fatigued.	The pH value was not measured.	First exercise was performed till fatigue, followed by 30 min break. Next five repetitions were performed for 24 s with a 216 s break in between. Participants hold upwards against gravity in a prone position.	Lumbar muscles	Intera, Philips, The Netherlands (PCr); NIRO-200, Hamamatsu Photonics KK (HHb, TOI)	During fatigue exercise PCr and TOI decreased whilst HHb increased. In the recovery period after 24 s of exercise, HHb levels decreased and PCr increased. The single measures reliability of the measurements of TOI and changes in TOI, PCr, and HHb ranged from good to excellent. The Tc had poor to fair reliability.
Hart et al., 2014 [47]	20, 22 ± 2, 10 female, 10 male	Constant-load submaximal plantar flexion	Decrease in pH from rest to end-exercise was 7.03 ± .08 to 6.86 ± .16	5 min exercise followed by 5 min recovery. 40% of individual maximum plantar flexion work-rate.	Gastrocnemius	Tim-Trio, Siemens Medical Solutions (PCr, ATP); Oxiplex TS, ISS (HHb)	PCr Tau: 33 ± 16 seconds; maximal ATP synthesis rate: 25 ± 9 mM/min; muscle reoxygenation rate: 48 ± 5 seconds.
Layec et al., 2014 [48]	20, 22 ± 2, 10 female, 10 male	Constant-load sub-maximal plantar flexion	pH dropped from 7.03 ± .08 at rest to 6.86 ± 16 at the end of exercise	After 1 min of data collection at rest, subjects exercised for 5 min followed by 5 min of recovery. Exercise intensity was set at 40% of MVC.	Gastrocnemius	Tim-Trio, Siemens Medical Solutions (PCr, ATP); Oxiplex TS, ISS (HHb)	At rest: PCr 32.6 ± 5.2 mM, ADP 9.5 ± 1.6 μM, TOI 68 ± 1%. At exercise end: PCr 18 ± 4 mM, ADP 43 ± 14 μM, TOI: 60 ± 8%
Bendahan et al., 2017 [49]	4, 37 ± 13, male	Protocol consisted of ramp, repeated isometric finger flexor performed at three different intensities (10, 20, 30% MVC). No resting period was performed. After a recovery period, a pressure cuff around the arm was rapidly inflated and was maintained for 6-min period.	Delta pH ranged from .02 ± .02 at 10% MVC to .21 ± .14 at 30% MVC.	Each step was performed in a 2-min trial and consisted of 80 total isometric contractions. An 8 min recovery followed the 3 exercise increments (10, 20, 30% MVC) Finally an ischemic period for 6 min was performed.	Superficial finger flexor	Bruker-Biospec 47/30, Bruker-Medical, Ettlingen, Germany (PCr, ATP); Oxymon, Artinis Medical Systems, Andelst, The Netherlands (HbO_2_)	Postischemic returning O_2_ does not impact any PCr recovery, since during the 6-min ischemia, PCr level has remained constant. During recovery, O_2_ recovery rate also does not appear to limit PCr recovery rate. NIRS shows O_2_ recovering with t1/2 of 0.4 min (24 s). PCr recovers after 30% MVC with a t1/2 of .27 min (16 s). Within the measurement errors, O_2_ and PCr show matching recovery kinetics.
Yanagisawa et al., 2017 [50]	7, 22 ± 2, male	Low-load ankle plantar flexion exercise (120 repetitions, 30% of one-repetition maximum) with and without BFR (130% of systolic blood pressure)	There was no difference in pH between the control and BFR conditions in the pre-exercise values, but differences in pH between the two conditions were observed at 2–4 min during exercise (at 2 min, *P* < 0.05; at 3 and 4 min, *P* < 0.01).	120 repetitions at 30% MVC.	Gastrocnemius	Signa Excite XIV, GE Healthcare, Milwaukee, WI, USA (PCr); HEM7420, OMRON, Kyoto, Japan (TOI)	TOI of both exercise conditions decreased during each exercise. BFR condition showed lower O_2_Hb and higher HHb values during exercise compared with the control condition. BFR condition showed greater Pi/PCr ratios during exercise than the control condition. In total, low-load BFR exercise stressed intramuscular high-energy phosphate metabolism greatly and created greater hypoxic and acidic environments within the exercising muscle in comparison with low-load non-restricted exercise.
Heskamp et al., 2020 [51]	20, 18–35, male	15 subjects performed continuous ankle dorsiflexion. 5 subjects performed intermitted isometric ankle dorsiflexion.	At the end of exercise pH range from 6.87–6.75	Continuous ankle dorsiflexion was performed at 30% MVC until exhaustion (2–5 min). Intermittent isometric ankle dorsiflexion started at 10% MVC and step wisely increased by 10% MVC every 30 sec until exhaustion (60–70% MVC).	Tibialis anterior	Sauter FL 500, Balingen, Germany (PCr); OxyMon MK III, Artinis Medical System, Elst, The Netherlands (O_2_Hb).	Following continuous isometric ankle dorsiflexion at 30% MVC until exhaustion, the recovery rate constants of O_2_Hb and of PCr, and the absolute rate of PCr recovery increased from distal to proximal along the length of the tibialis anterior. As PCr resynthesis is proportional to the oxygen-dependent suprabasal ATP synthesis, and as additionally Hb recovers faster proximally than distally, this indicates that O_2_ supply also exhibits a proximo-distal gradient with the highest O_2_ supply proximally. Correlation analysis revealed that PCr recovery constant correlated strongly with O_2_Hb recovery constant (*r* = .956, *p* = .011).

*AO* arterial occlusion, *BFR* blood flow restriction, *HbO*_*2*_*/O*_*2*_*Hb/oxy-Hb/Oxyhemoglobin* oxygenated hemoglobin/myoglobin, *HHb/deoxy-Hb* deoxygenated hemoglobin/myoglobin, *HbO*_*2*_*Tc* time constant for oxygenated hemoglobin/myoglobin, *Hb-Mg* hemoglobin and myoglobin, *Hb* hemoglobin, *Hb*_*tot*_ total hemoglobin, *∆HbO*_*2*_ change in oxygenated hemoglobin, *∆Hb*_*tot*_ change in total hemoglobin, *mVO*_*2*_ muscle oxygen consumption, *PCr* phosphocreatine, *Pi* phosphate, *PCrTc* time constant for phosphocreatine, *Reoxy-rate* Rate of oxygenated hemoglobin/myoglobin increase, *Tau* time constant when value has reached 63% of the resting value, *Tc* time constant, *TOI* tissue oxygenation, *VO*_*2mus*_ muscle oxygen consumption measured with deoxygenation rate of hemoglobin and myoglobin, *VIB* vibration

### Quality of studies

EPHPP tool was conducted to assess the cross-sectional and intervention studies. The complete analysis is reported in Table 2. The mean quality score for the 28 articles was 2.15 ± 0.23, thereby moderate. All articles achieved a moderate score. The withdrawals and dropouts category did not apply to most studies because a lower dropout risk exists in crossover study designs. Studies have been rated between weak and strong when other techniques were applied.

**Table 2:** Quality assessment based on the Effective Public Health Practice Project (EPHPP)

Author	Selection Bias	Study Design	Control for Confounders	Blinding	Data Collection	Withdrawals and Dropouts	Global Rating	Global Rating
McCully et al. [22]	2	1	2	2	3	N/A	2	Moderate
Hamaoka et al. [26]	2	1	2	2	3	N/A	2	Moderate
Yoshida et al. [27]	2	2	2	2	2	1	1.83	Moderate
Binzoni et al. [28]	2	1	1	2	3	N/A	1.80	Moderate
Binzoni et al. [29]	2	1	3	2	3	N/A	2.20	Moderate
Boushel et al. [30]	2	1	2	2	2	N/A	1.80	Moderate
Kutsuzawa et al. [31]	2	1	2	2	3	N/A	2	Moderate
Sako et al. [32]	2	1	2	2	3	N/A	2	Moderate
Hamaoka et al. [33]	2	1	2	2	3	N/A	2	Moderate
Kime et al. [34]	2	1	2	2	3	N/A	2	Moderate
Kime et al. [35]	2	1	2	2	3	N/A	2	Moderate
Nagasawa et al. [36]	3	1	2	2	3	N/A	2.2	Moderate
Forbes et al. [37]	3	2	2	2	3	N/A	2.4	Moderate
Kimura et al. [38]	3	3	2	2	2	N/A	2.4	Moderate
Jones et al. [39]	3	2	2	2	3	3	2.4	Moderate
Forbes et al. [40]	3	2	2	2	3	3	2.5	Moderate
Zange et al. [41]	3	1	2	2	3	N/A	2.2	Moderate
Layec et al. [42]	3	1	2	2	3	1	2	Moderate
Willcocks et al. [43]	2	1	2	2	3	2	2	Moderate
Layec et al. [44]	3	2	1	2	3	1	2	Moderate
Layec et al. [45]	3	2	2	2	3	3	2.5	Moderate
Ryan et al. [6]	3	1	3	2	3	3	2.5	Moderate
Fulford et al. [46]	3	2	2	2	3	1	2.2	Moderate
Hart et al. [47]	3	2	2	2	3	3	2.5	Moderate
Layec et al. [48]	3	2	2	2	3	3	2.5	Moderate
Bendahan et al. [49]	3	1	2	2	3	1	2	Moderate
Yanagisawa et al. [50]	3	1	2	2	3	N/A	1.8	Moderate
Heskamp et al. [51]	3	1	1	2	3	2	2	Moderate

All questions have been rated as strong (3 points), moderate (2 points) or weak (1 point), and domain scores were averaged to provide the total score. The maximum total score per study was 3.00. Based on their total score, studies were assigned a quality rating of weak (1.00–1.50), moderate (1.51–2.50) or strong (2.51–3.00)

## Discussion

Our systematic review of 28 studies identified research investigating energy-rich phosphate changes and NIRS during rest, exercise, and ischemia. All articles achieved a moderate score (Range = 1.80–2.50). Since the relationship between two physiological variables were compared in the studies, most included studies assessed the variables within a cross-sectional study design. This led 18 out of 28 studies to a low grading concerning the study design. Although observational study design is appropriate to infer a relationship [53], the quality appraisal tool tended to produce lower quality scores for those studies.

Overall, the results of this review were inconclusive. No consistent relationship between oxygen levels and PCr were identified. Measurement protocols were heterogeneous and had a low sample size. Additionally, heterogeneous formulas for NIRS-based variables were used, making it challenging to compare oxygen kinetics between studies. Because of the high heterogeneity of NIRS-based variables, the categorization of variables was not applied. Nevertheless, considering the current state of knowledge, the application of NIRS to determine changes in energy-rich phosphates would appear appropriate when certain conditions are met.

### Energy rich phosphates and NIRS at rest with and without AO

Differences in oxygen kinetics and the time course of high-energy phosphates during rest with AO have been observed. In general, the absence of blood flow with an AO was used to decouple muscle oxygen delivery from oxygen consumption and focus on muscle oxygen consumption. Hamaoka et al. [26] described the first observations at rest. Muscle oxygen declines in a linear fashion attenuating at 240 sec and leading to a plateau at 360 sec. Particularly at rest, these observations can vary with low metabolic rate and high individual variation; for this reason, muscle activation is included in the protocol to stimulate metabolism. When O_2_ was measured to be insufficient to maintain ATP level, PCr decreased, broken down to maintain ATP level for resting metabolism [26]. This can only be examined if a sufficient gradient is reached between blood vessels and the tissue or mitochondria. Furthermore, Sako et al. [32] observed a decrease in PCr as soon as Hb reached a plateau in AO resting measurements 5 min after the start of AO. Notably, when AO was applied for 5 min periods during rest in the study by Binzoni & Hildebrand [28], PCr and ATP concentrations did not change. In contrast to the constant PCr and ATP levels at rest, a constant change in desaturation was observed during the applied ischemic periods. It can be concluded that at rest under an extended period of ischemia (i.e., no longer than 5 min), no change in PCr occurs, even if changes in NIRS-related values are observed. This can be extrapolated because if sufficient oxygen levels are available to support oxidative metabolism, high resting PCr can be maintained. As oxygen levels decline, this is no longer the case. Therefore, during short periods of ischemia at rest, NIRS will not reflect PCr.

The deoxygenation rate of NIRS-O2 has been shown to be a function of work rate [54], and during ischemia at rest and exercise, 2 to 8-fold increases have been documented [6, 30]. Since pH in skeletal muscle decreases only when coupled to muscular contraction, the glycolysis rate is assumed to be low at rest [55] and increases exponentially with the onset of exercise. Hence, the authors attempted to explain that oxygen stores, namely Hb and Mb, could have the capabilities to maintain tissue metabolism during AO at rest [30].

### Energy-rich phosphates and NIRS during exercise

A few studies measured NIRS and MRS simultaneously during exercise. Without AO rate of deoxygenation (time constant: 42 ± 12.5 sec) was in line with the time constant of the decrease in PCr (48.2 ± 10.2 sec) at the onset of intermitted contraction exercise (5 sec isometric contractions and 5 sec relaxation) at 50% of MVC [33]. Additionally, to show that at 50% of MVC exercise can potentially occlude arterial blood flow, Hamaoka et al. [33] compared a 10 sec occluded isometric hold with a non-occluded trial. The rate of deoxygenation was similar in both trials (2.23 ± 1.25%/sec versus 2.13 ± 1.18%/sec), hence in their preliminary study, Hamaoka and colleagues [33] were the first that demonstrates that the rate of muscle deoxygenation by NIRS during an intermittent exercise at 50% of MVC without AO indicates only muscle oxygen consumption.

The biochemical effect of extensive ATP hydrolysis from exercise resulting in increasing proton accumulation and consequently a shift in pH [27, 37, 47, 48, 56] is an important factor to consider when looking at NIRS and PCr response during exercise. In a study by Willcocks and colleagues [43] using constant-work rates without pH changes, HHb and PCr were not associated; PCr decreased from rest to end-exercise regardless of HHb responses. However, when exercise intensity increases and thus significant changes in pH occur, ATP production increased approximately twofold when MVC was elevated from 15 to 30% in rhythmic handgrip exercise when using either MRS or NIRS data to calculate high energy phosphates [30]. In a ramp test, energy-rich phosphate changes and HHb show similar behavior [49]. During 10, 20, and 30% of MVC, NIRS data can predict the course of PCr [49]. Nevertheless, no direct comparison between PCr and HHb was undertaken. From a physiological perspective, at higher intensities above 50% of MVC, an increased glycolytic rate could decrease the ADP effect on oxidative phosphorylation and, therefore, the availability of intramuscular O_2_ in mitochondria [37, 38, 46]. Accordingly, when lower intensity exercise is performed (e.g., at 30% of MVC), intramuscular oxidative metabolism matters more and curtails both PCr and glycolysis as energy suppliers [31, 38, 44]. Thus, visual inspection of data, quantitative analysis, and physiological explanations support the assumption that exercise intensity similarly impacts the rate of decline of muscle oxygenation and PCr. Considering this fact, we support the hypothesis that the change of PCr is greatly influenced oxygen availability [8–10].

PCr and muscle oxygen kinetics can be affected by prior ‘priming’ exercise, dependent on intensity. In particular, shifts in pH influence PCr kinetics [57]. PCr recovery follows a biexponential function with two distinct phases, an initial and a slow component [58]. While it is unclear what the exact mechanisms of control (i.e., cytosolic ADP) of the initial phase are, the slow component would appear to be tightly coupled to pH. This is a reasonable outcome considering the role of H+ in creatine kinase equilibrium and, therefore, the mass-action ratio. Priming can increase muscle oxygenation in a subsequent high intensity exercise bout with pH level and muscle efficiency influencing PCr hydrolysis. This may lead to a larger reduction in PCr [39]. Though an increase in muscle oxygen could be seen, PCr decreased faster and thereby dissociated PCr kinetics from muscle oxygen kinetics. Forbes and colleagues [40] examined the influence of the recovery time between two high intensity exercise bouts with 3 min, 6 min, and 15 min. PCr slow component was similarly reduced in all three bouts, whereas HHb onset kinetics were only slowed in the 3 min bouts. During the mid to later (approx. 2–6 minute) course of the second exercise bout, independent of the recovery time, PCr was significantly higher than in the first, indicating changed metabolism regardless of the recovery time. HHb was elevated only in the exercise bout after 3 min recovery. As a result, this led to the assumption that PCr consumption is not influenced by O_2_ delivery, fatigue mechanisms, PCr level or intracellular acidosis at the onset of the second exercise bout [40].

Contrastingly, Layec et al. [42] reported an increased deoxygenation amplitude, a decrease in PCr breakdown, and a decrease in glycolysis in consecutive bouts of high-intensity knee-extension exercise, illustrating a shift from glycolytic to oxidative ATP production in the second exercise bout. A reduced ADP stimulus was proposed as the reason for the shift since, at the end of the second exercise bout oxygenation level was increased, and the ADP level decreased. This could indicate higher intracellular O_2_ extraction for higher ATP production rates with concurrent lower ADP levels [42]. Although a coupling of NIRS data with PCr behavior was observed in this study, the authors argued that the control of oxidative phosphorylation could rely on the more complex relationship between PCr and muscle oxygen kinetics and essentially be influenced by additional factors such as phosphate potential, pH, and ADP [42]. Given these contradictions, there appears to be room for interpretation regarding how exercise and priming influence muscle oxygen kinetics and phosphate metabolism.

Further, when exercise is introduced under AO, metabolic stress is indicated by both PCr and NIRS parameters [41, 50]. Blood flow restriction intervention from Yanagisawa et al. [50] revealed that both PCr and Hb were significantly changed when AO occurred. Moreover, during the four-minute course of the exercise, the comparison of restricted versus non-restricted blood flow constituted a significant difference in the decrease of Hb from the very first minute onward; in fact, PCr decreases were significantly different from the second minute onward. These findings support the assumptions that insufficient O_2_ supply as a result of AO occurs, ATP supply could increasingly depend on oxygen stores and glycolytic metabolism. Nevertheless, the study did not directly compare the course of Hb and PCr and, thus, limits further conclusions.

### Energy rich phosphates and NIRS during recovery

Most studies in the present review included recovery measurements. McCully et al. [22] were the first to assess the relationship between PCr and Hb saturation. Their results indicated a similar course of PCr and Hb after exercise. Additionally, their results demonstrated a decoupling of Hb and PCr when exercise intensity was increased to maximal bouts intensity [22]. Consequently, the potential benefit of the function of reoxygenation kinetics as an indicator of PCr recovery kinetics during recovery periods may be limited to low intensity exercise with controlled pH (approximate pH of 7.0).

Moreover, in high oxidative capacity muscle, it was shown that after a short duration, high intensity isometric exercise, the reoxygenation rate during the recovery phase is slower due to greater O_2_ utilization capacity [34, 35]. In particular, these observations were made when O_2_ demand was maximized at high intensity exercise. Results of Kime et al. [34, 35] and Hamaoka et al. [33] show, contrary to McCully et al. [22], that higher intensities are necessary to create a bridge between NIRS and MRS-based PCr. This is likely due to the degree to which NIRS is removed from the direct measurement of PCr, and oxygen dynamics functions as a limited surrogate. It was shown that muscle oxygen consumption at 90% of MVC was significantly higher than at 30 and 60% of MVC. Additionally, the correlation between Tc of PCr and HbO_2_ reoxygenation rate could only be found at 90% of MVC and not at 30 and 60% of MVC [35]. Consequently, at higher intensities above approximately 50% of MVC, an increase in glycolytic metabolism could decrease the ADP effect on oxidative phosphorylation and, therefore, the availability of intramuscular O_2_ in mitochondria [38].

There may be room here for a confounder in the relationship between PCr breakdown and recovery, and muscle oxygenation kinetics, which may clarify some of the contradiction. Difference in muscle oxygen on and off-set kinetics as a result of fitness may be the factor determining the goodness of fit between muscle oxygenation and PCr [22]. The higher the fitness the smaller the O_2_ supply and demand gap at the onset and offset of exercise, as shown by VO_2_ kinetics [59], and therefore the better the relationship between deoxygenation and reoxygenation and PCr kinetics. Finally, Heskamp and colleagues [51] showed that the close relation between PCr and oxygen supply remains independent of spatial variation. The oxygen supply increased from distal to proximal along the tibialis anterior muscle, just as the PCr recovery rate did. A strong correlation between Hb recovery rate constant and PCr recovery rate constant confirmed the relationship (*r* = .956 and *r* = .852).

A calculation of ATP change based on NIRS variables was demonstrated by Hamaoka et al. [26] and Sako et al. [32]. Hamaoka et al. [26] and Sako et al. [32] calculated muscle metabolic rate based on NIRS values and expressed it as millimolar ATP per second. Significant correlation occurred between NIRS-based muscle O_2_ consumption in absolute terms with ADP and PCr [26] and between NIRS-based muscle oxidative metabolic rate with PCr resynthesis rate post exercise [32]. The results provide evidence that NIRS could quantitively indicate the rate of muscle oxidative metabolism. The calculation of muscle consumption and muscle oxidative metabolic rate in both studies was similarly achieved *(Hb decline rate with AO after exercise)/(Hb rate with AO at rest) × (Resting metabolic rate measured by P-MRS).* The equation illustrates the dependence of these methods on *P*-MRS data. In the absence of *P*-MRS data, NIRS data alone cannot complete these calculations therefore limiting their applications.

In contrast, recovery constants only by NIRS have been experimentally validated against *P*-MRS independent of *P*-MRS [6, 36]. These time constants correlated well (*r*2 = .88–.95) and agreed in Bland-Altman Plots. The authors demonstrated that the indices solely based on NIRS could provide information about the muscle oxidative capacity.

### Limitations

The present study has two major limitations. First, many of the studies evaluated applied time constants or complex calculated indices to provide information about muscle metabolism rather than to compare the direct course of PCr and muscle oxygen kinetics during exercise. The heterogeneous data offered in the review provides evidence for a relationship between PCr and muscle oxygen kinetics when appropriate protocol settings are used, as correlations confirm (Table 1). Essential conditions for study protocols are measurement methods, intensity of priming exercise, recovery time, and intracellular pH level. Thus, comparison and interpretation of the results are strongly limited. Second, this study aimed to obtain information about the application of NIRS to examine integrated energetics in athletes. However, NIRS can only measure the balance between O_2_ consumption and delivery. This limits its scope of application, and the authors mainly cited used the AO to calculate oxygen consumption and derive from that phosphate levels. AO restricts the practical application of NIRS to be integrated into dynamic measurement and diagnostics. Hamaoka et al. [33] were the only ones to examine the necessity of AO above 50% of MVC. Based on their assumptions, it could be possible to solely measure the muscle oxygen consumption with high intensity exercises without AO and, thus, indicate the course of PCr during everyday sport science settings. Because of the significant correlations between PCr and the rate of deoxygenation (*r =* .96, *p* > .05) and the agreement of the time constant of the rise of the rate of deoxygenation and the time constant of the decrease in PCr, it seems that NIRS has the potential to indicate PCr during exercise. Though first quantitative measures were provided, further investigations would be beneficial to support those findings and strengthen the evidence of muscle oxygen kinetics as an indicator of energy rich phosphates in an applied sport science context.

### Future directions

In future studies a greater emphasis should be placed on the integration of NIRS with *P*-MRS to leverage the strengths of both measurements. Exercise, in particular strenuous exercise, involves low oxygen conditions and NIRS is uniquely placed to characterize oxygen levels during such exercise. *P*-MRS on the other hand is uniquely designed to measure changes in glycolysis and muscle pH, which is important in understanding strenuous exercise. While not covered in this review, joining NIRS measures with other MRS or MRI techniques like 1H-MRS may provide a more comprehensive evaluation of muscle energetics, by exploiting the different techniques inherent strengths and weaknesses [60–62]. In this way future studies can be classified into three general categories, those that include NIRS as a necessity for context with MRS and MRI data collection, those that include NIRS as an extension to the findings of the MRS/MRI findings, and those that apply NIRS independently or as surrogate of MRS/MRI findings. While true for all three categories, the third category in particular lends itself to combining NIRS and other noninvasive assessments to answering very practical and in situ questions, given the increasing portability of NIRS and other noninvasive tools. For example, the combination of NIRS to assess muscle metabolism and triaxial accelerometry to measure muscle movements and position in space; or with electromyography (EMG) to measure muscle activation and metabolism. Including a multi-site NIRS measurements with assessments of whole-body oxygen consumption would seem to greatly advance our understanding of whole body movement and metabolism. In summary, muscle responses to exercise are a fundamental part of the how the body addresses questions exercise and intensity, and future research should include both comprehensive assessments of muscle function with multimodal approaches, as well as assessments of local and whole body responses to exercise.

## Conclusion

In conclusion, 28 studies have included both NIRS derived muscle oxygen kinetics and energy rich phosphates. The results suggest that the application of NIRS can indicate the change in energy rich phosphates when assessed in an appropriate protocol setting. High intensity exercise may be necessary, such that oxygen delivery is disrupted. For this reason, the AO method or high intensity exercise should be included in NIRS-based studies evaluating high energy phosphates. The heterogeneity of the data, protocols and interoperations limit the interpretation of the data. More research needs to be conducted using MRS and NIRS without AO and with high intensity exercise to demonstrate an agreement in the concurrent course of muscle oxygen kinetics and muscle energetics.

## Data Availability

All data generated or analysed during this study are included in this published article.

## Abbreviations

*AO*: Arterial occlusion
*BFR*: Blood flow restriction
*HbO*_*2*_*/O*_*2*_*Hb/oxy-Hb/Oxyhemoglobin*: Oxygenated hemoglobin/myoglobin
*HHb/deoxy-Hb*: Deoxygenated hemoglobin/myoglobin
*HbO*_*2*_*Tc*: Time constant for oxygenated hemoglobin/myoglobin
*Hb-Mg*: Hemoglobin and myoglobin
*Hb*: Hemoglobin
*Hb*_*tot*_: Total hemoglobin
*∆HbO*_*2*_: Change in oxygenated hemoglobin
*∆Hb*_*tot*_: Change in total hemoglobin
MRI: Magnetic resonance imaging
MRS: Magnetic resonance spectroscopy
*mVO*_*2*_: Muscle oxygen consumption
*NIRS*: Near-infrared spectroscopy
PCr: Phosphocreatine
*PCrTc*: Time constant for phosphocreatine
*Pi*: Inorganic phosphate
*P-MRS*: Phosphorus nuclear magnetic resonance spectroscopy
*Reoxy-rate*: Rate of oxygenated hemoglobin/myoglobin increase
*Tau*: Time constant when value has reached 63% of the resting value
*Tc*: Time constant
*TOI*: Tissue oxygenation
*VO*_*2mus*_: Muscle oxygen consumption measured with deoxygenation rate of hemoglobin and myoglobin
*VIB*: Vibration
